# Action recognition using Natural Action Structures

**DOI:** 10.1186/1471-2202-13-S1-P18

**Published:** 2012-07-16

**Authors:** Xiaoyuan Zhu, Zhiyong Yang, Joe Z Tsien

**Affiliations:** 1 Brain and Behavior Discovery Institute, Georgia Health Sciences University, Augusta, Georgia, 30912, USA; 2 Department of Neurology, Georgia Health Sciences University, Augusta, Georgia, 30912, USA; 3 Department of Ophthalmology, Georgia Health Sciences University, Augusta, Georgia, 30912, USA

## 

Humans can detect, recognize, and classify natural actions in a very short time. How this is achieved by the visual system and how to make machines understand human actions have been the focus of neuro-scientific studies and computational modeling in the last several decades. A key issue is what spatial-temporal features should be encoded and what the characteristics of their occurrences are in natural actions. We propose a novel model in which Natural Action Structures (NASs) (see Figure 1), i.e., multi-size, multi-scale, spatial-temporal concatenations of local features, serve as the basic encoding units of natural actions. In this concept, any action is a spatial-temporal concatenation of a set of NASs, which convey a full range of information about natural actions. We took several steps to extract and identify these structures and selected a set of informative natural action structures to classify a range of human actions. We found that the NASs obtained in this way achieved a significantly better recognition performance than low-level features [1] and that the performance was better than or comparable to the best current models (see Table 1).

**Figure 1:**
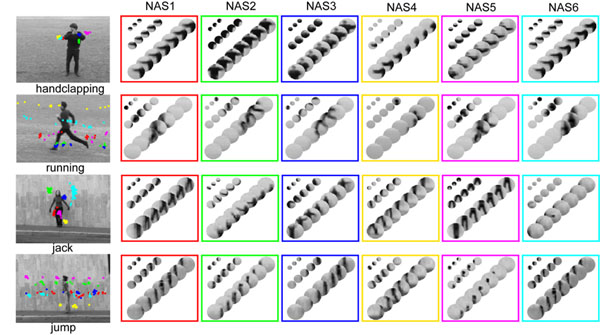
Examples of NASs. 6 frequent NASs compiled from each of the 4 actions in the KTH and the Weizmann dataset. The locations of the NASs in the videos and the NASs are indicated by the same color.

**Table 1:** 

Methods	KTH	Weizmann
**NASs**	92.7%	96.7%
**Cuboids**	88.5%	94.4%
**Yao et al. **[2]	92.0%	95.6%
**Niebles et al. **[3]	83.3%	90.0%

## Conclusions

NASs contain a variety of information about human actions and are robust against variations due to noises, occlusions, changes in scales, and a range of structural changes since they are concatenations of features at multiple spatial-temporal scales. The results suggest that NASs can be used as the basic encoding units of human actions and activities and may hold the key to the understanding of human ability of action recognition.
